# Darunavir-cobicistat versus lopinavir-ritonavir in the treatment of COVID-19 infection (DOLCI): A multicenter observational study

**DOI:** 10.1371/journal.pone.0267884

**Published:** 2022-05-04

**Authors:** Eman Zeyad I. Elmekaty, Rim Alibrahim, Rania Hassanin, Sitelbanat Eltaib, Ahmed Elsayed, Fatima Rustom, Mohamed Izham Mohamed Ibrahim, Mohammed Abu Khattab, Hussam Al Soub, Muna Al Maslamani, Abdullatif Al-Khal

**Affiliations:** 1 Communicable Diseases Center, Hamad Medical Corporation, Doha, Qatar; 2 Clinical Pharmacy & Practice Department, College of Pharmacy, QU Health, Qatar University, Doha, Qatar; Banaras Hindu University Institute of Medical Sciences, INDIA

## Abstract

**Background:**

Coronavirus Disease 2019 (COVID-19) is an evolving pandemic that urged the need to investigate various antiviral therapies. This study was conducted to compare efficacy and safety outcomes of darunavir-cobicistat versus lopinavir-ritonavir in treating patients with COVID-19 pneumonia.

**Methods and findings:**

This retrospective, multicenter, observational study was conducted on adult patients hospitalized in one of the COVID-19 facilities in Qatar. Patients were included if they received darunavir-cobicistat or lopinavir-ritonavir for at least three days as part of their COVID-19 treatments. Data were collected from patients’ electronic medical records. The primary outcome was a composite endpoint of time to clinical improvement and/or virological clearance. Descriptive and inferential statistics were used at alpha level of 0.05. A total of 400 patients was analyzed, of whom 100 received darunavir-cobicistat and 300 received lopinavir-ritonavir. Majority of patients were male (92.5%), with a mean (SD) time from symptoms onset to start of therapy of 7.57 days (4.89). Patients received lopinavir-ritonavir had significantly faster time to clinical improvement and/or virological clearance than patients received darunavir-cobicistat (4 days [IQR 3–7] vs. 6.5 days [IQR 4–12]; HR 1.345 [95%CI: 1.070–1.691], P = 0.011). Patients received lopinavir-ritonavir had significantly faster time to clinical improvement (5 days [IQR 3–8] vs. 8 days [IQR 4–13]; HR 1.520 (95%CI: 1.2–1.925), P = 0.000), and slower time to virological clearance than darunavir-cobicistat (25 days [IQR 15–33] vs. 21 days [IQR 12.8–30]; HR 0.772 (95%CI: 0.607–0.982), P = 0.035). No significant difference in the incidence or severity of adverse events between groups. The study was limited to its retrospective nature and the possibility of covariates, which was accounted for by multivariate analyses.

**Conclusion:**

In patients with COVID-19 pneumonia, early treatment with lopinavir-ritonavir was associated with faster time to clinical improvement and/or virological clearance than darunavir-cobicistat. Future trials are warranted to confirm these findings.

**Trial registration:**

ClinicalTrials.gov number, NCT04425382.

## Introduction

Novel Coronavirus Disease 2019 (COVID-19) was first emerged in Wuhan, China, at the end of 2019, resulting in a pandemic crisis [1, 2]. It is caused by Severe Acute Respiratory Syndrome Coronavirus 2 (SARS-CoV-2) virus, that spread rapidly to other countries resulting in more than 150 million confirmed cases and over three million deaths worldwide [3]. The estimated global mortality rate is more than 5.7% posing a significant threat to global health [4]. As of May 01, 2021, there were 206,302 positive cases, 14,766 active cases under treatment and 465 deaths in the country [3].

The spectrum of the infection ranges from mild, self-limiting respiratory symptoms to severe progressive pneumonia, acute respiratory distress syndrome (ARDS) requiring Intensive Care Unit (ICU) admission, and eventually death [5–7]. Numerous candidate agents have been investigated for the treatment of COVID-19 in previous studies at different parts of the world with inconclusive outcomes [8]. Protease inhibitors, developed to treat HIV infections, were studied as potential agents due to their in vitro inhibitory activity against SARS-CoV, Middle East Respiratory Syndrome coronavirus, and SARS-CoV-2 [9–12].

Many studies were conducted to evaluate the safety and efficacy of various protease inhibitors in COVID-19 patients, with lopinavir-ritonavir being the most commonly investigated agent followed by darunavir-cobicistat [13]. However, their use was limited because of side effects and significant drug interactions, mainly due to the inhibition of hepatic cytochrome P450 3A4 and p-glycoprotein [14, 15]. In a report from South Korea, lopinavir-ritonavir showed some efficacy in a patient with COVID-19 [16]. In contrast, in another trial of patients with severe COVID-19, no statistically significant difference was observed in the time to clinical improvement compared to the standard of care group [17].

Darunavir-cobicistat, at high concentration, was also associated with in vitro inhibition of SARS-CoV-2 [12]. It has better safety and tolerability profile than lopinavir-ritonavir [18]. Compared to ritonavir, cobicistat had a lower potential for undesirable drug-drug interactions and a better safety profile [19]. Thus, its efficacy and safety were evaluated in a small pilot study of patients with COVID-19 pneumonia with no significant outcomes [20].

Since the start of the pandemic, multiple organizations and healthcare institutions developed guidelines for the management of patients with COVID-19 infection. These guidelines were continuously updated as new scientific knowledge and research findings emerge [21–24]. In Qatar, we have fifteen versions of treatment guidelines for COVID-19 infection and these guidelines had dramatic changes based on the latest local data and evidence-based recommendations.

Up to our knowledge, no head-to-head study compared darunavir-cobicistat versus lopinavir-ritonavir for treatment of COVID-19 infection. Therefore, this study was conducted to compare darunavir-cobicistat versus lopinavir-ritonavir’s efficacy and safety outcomes in the treatment of patients with COVID-19 pneumonia.

## Materials and methods

### Study design

This was a retrospective, multicenter, observational study design, comparing the outcomes of patients who received either darunavir-cobicistat (Rezolsta^®^ [800mg Darunavir/ 150mg Cobicistat] 1 tablet orally once daily) or lopinavir-ritonavir (Kaletra^®^ [200mg Lopinavir/ 50mg Ritonavir] 2 tablets orally twice daily) as part of their COVID-19 management according to the national treatment guideline in Qatar.

### Ethical consideration

The study was approved by the Institutional Review Board at Hamad Medical Corporation (HMC) Medical Research Center (MRC# 05–069) and registered at ClinicalTrials.gov (NCT04425382). The study was granted a waiver of documentation of consent, in which research information sheets were provided to patients/family members for data collection. No additional administrative permissions were required to access the raw data. All data used in this study were fully anonymized before their use.

### Study location and timeline

The study was conducted at HMC, the principal public healthcare organization that provides care to all COVID-19 patients in the State of Qatar. It provides secondary and tertiary care for hospitalized patients in thirteen hospitals across the country. The study was carried out between 1^st^ March 2020 and 29^th^ April 2020.

### Study population and sampling method

The study population include hospitalized patients who were 18 years of age or older, with laboratory-confirmed COVID-19 infection, with radiological evidence of pneumonia, and received at least three days of either darunavir-cobicistat or lopinavir-ritonavir as part of the treatment regimen for COVID-19 pneumonia. The use of darunavir-cobicistat and lopinavir-ritonavir was implemented as a standard-of-care in the country and the selection of a particular regimen was made at the discretion of the treating physician.

Diagnosis of COVID-19 infection done by positive RT-PCR assays from nasopharyngeal/oropharyngeal respiratory samples using TaqPath COVID-19 Combo Kit (Thermo Fisher Scientific, Waltham, Massachusetts) or Cobas SARS-CoV-2 Test (Roche Diagnostics, Rotkreuz, Switzerland). Pneumonia was defined as the presence of infiltrate, ground-glass or patchy opacities, or consolidation on the chest x-ray or CT scan imaging.

At the time of the study, treatment regimen for COVID-19 pneumonia in the national guideline included supportive care, chloroquine/hydroxychloroquine, azithromycin, oseltamivir, protease inhibitors, antibiotics, and/or ribavirin. Steroids, pegylated-interferon a2a, or tocilizumab can be added for those with severe disease not responding to other treatment modalities, has evidence of significant systemic inflammation, ARDS, and/or septic shock with evidence of cytokine release syndrome. Regimens were individualized based on the severity of the disease. The intended duration of protease inhibitors as per the treatment protocol was 14 days. No exclusion criteria were applied in this study. All patients admitted in one of the COVID-19 facilities and fulfilled the inclusion criteria were included.

### Outcome measures

The study’s primary outcome was a composite endpoint of time to clinical improvement and/or virological clearance up to 90 days. Clinical Improvement was defined as the time to normalization of fever (defined as temperature <37.8°C for 72 hours) and/or the resolution of baseline sign and symptoms without the need for symptomatic treatment. Virological clearance was defined as the time to two consecutive negative and/or inconclusive COVID-19 PCR results. These endpoints of clinical improvement and virological clearance were used in previous COVID-19 studies, and the definitions were previously recommended in the World Health Organization (WHO) guideline [25–29]. This study was conducted before the release of the recommended outcome measures for COVID-19 clinical research by the WHO COVID-19 management working group [30].

Secondary outcomes included virological clearance at day 14, day 21, and day 28, clinical deterioration (defined as the need for respiratory support, vasopressor use, corticosteroid/immunomodulation therapy use, or prone positioning), the incidence of adverse events as assessed by the Common Terminology Criteria for Adverse Events (CTCAE) version 5.0 [31], development of ARDS as per Berlin Definition [32], length of hospital-stay, all-cause mortality at 30-days, and the rate of premature discontinuation of study treatment.

### Data collection procedure

Data were collected from patient’s electronic medical records (Cerner Millennium® Software) by the research investigators and independently validated by different investigators to ensure the accuracy and consistency of the collected data. Variables collected including patients’ demographics, clinical, radiological, and laboratory data.

For baseline signs and symptoms, the onset of symptom date was defined as the day when the first symptom was noticed. The date of resolution of symptoms was defined as the first date without symptoms or the need for symptomatic treatment. The patients’ full medical history, comorbidities, medications details were collected. Electrocardiograms were reviewed to assess QTc intervals at baseline and after starting therapy. Safety data pertaining to the treatment adverse drug reactions (ADRs) and the reasons for premature discontinuation of therapy were also collected. Premature therapy discontinuation was defined as receiving <75% of the planned treatment duration (<11 days). Clinical deterioration was considered an outcome of the study therapy if it occurred ≥ two days from starting protease inhibitors.

### Statistical analysis

Data were gathered in Excel program. All statistical analyses were done using the statistical package, SPSS version 26 (Armonk, NY: IBM Corp.). Descriptive statistics have been used to summarize patient’s characteristics. Categorical data were expressed by frequency (percentage), while continuous values were expressed as mean ± SD or median and interquartile range (IQR). Data normality was tested using Kolmogorov-Smirnov test. The means of two groups were examined with the Mann–Whitney U or independent t-test (depends on normal distribution of data) and categorical data was analyzed with the chi-square or Fisher’s exact tests (as appropriate). The clinical progressions, i.e. the time to clinical improvement and virological clearance were presented by Kaplan–Meier plot and the difference was compared using a log-rank test. The hazard ratios with 95% confidence intervals were calculated using the Cox proportional-hazards model, which allows other explanatory variables (covariates) to be consideration. A two-sided p-value of <0.05 was considered statistically significant.

## Results

### Patients’ characteristics

A total of 517 patients were screened, and 400 patients met the eligibility criteria and included in the analysis 100 (25%) patients in the darunavir-cobicistat group and 300 (75%) patients in the lopinavir-ritonavir group). The majority of the patients were male (n = 370, 92.5%), with a mean age of 45.80 years (SD ±12.26). Half of the study population (n = 215, 53.8%) were previously healthy and had no comorbidities, with 85.8% (n = 343) of the patients has normal oxygen saturation at baseline. Study therapy was started within seven days of symptoms onset in 56.6% of the patients.

Table 1 summarized baseline demographic and clinical characteristics of the two groups. Patients in the lopinavir-ritonavir group had younger age (p = 0.006) and fewer comorbidities (p = 0.010) compared with patients in the darunavir-cobicistat group. Around half of the patients who received darunavir-cobicistat received ribavirin therapy (48% vs 7.3%, p = 0.001). Fever, cough, shortness of breath were the most common presenting symptoms in both treatment arms.

**Table 1:** Baseline characteristics of the study population.

	Characteristic	Total	Lopinavir-Ritonavir (n = 300)	Darunavir-Cobicistat (n = 100)	p-value
**Demographic Data**					
	Gender				
	Male	370 (92.5)	280 (93.3)	90 (90)	0.273
	Age (years)	45.8 ± 12.3	44.7 ± 11.4	49.1 ± 14.2	0.006
	Age group				0.011
	<60 Years	333 (83.3)	258 (86)	75 (75)	
	≥ 60 Years	67 (16.8)	42 (14)	25 (25)	
	Region of origin				0.000
	South Asia	296 (74)	236 (78.7)	60 (60)	
	Middle East	77 (19.3)	44 (14.7)	33 (33)	
	East Africa	14 (3.5)	12 (4)	2 (2)	
	Europe	8 (2)	7 (2.3)	1 (1)	
	America	5 (1.3)	1 (0.3)	4 (4)	
	Smoking status				0.613
	Smoker	258 (64.5)	192 (64.0)	66 (66)	
	Ex-smoker	20 (5.0)	13 (4.3)	7 (7)	
	Never smoked	28 (7.0)	21 (7)	7 (7)	
	Unknown	94 (23.5)	74 (24.7)	20 (20)	
**Clinical Data**					
	Documented fever	308 (77.0)	235 (78.3)	73 (73)	0.272
	Symptomatic at baseline	368 (92.0)	275 (91.7)	93 (93.0)	0.670
	Fever	366 (91.5)	276 (92.0)	90 (90.0)	0.535
	Cough	350 (87.5)	264 (88.0)	86 (86.0)	0.600
	Sore throat	132 (33.0)	100 (33.3)	32 (32.0)	0.806
	Runny nose	34 (8.5)	25 (8.3)	9 (9.0)	0.836
	Chest pain	31 (7.8)	23 (7.7)	8 (8.0)	0.914
	Shortness of breath	164 (41)	114 (38.0)	50 (50.0)	0.035
	Nausea/Vomiting	53 (13.3)	34 (11.3)	19 (19.0)	0.050
	Diarrhea	32 (8.0)	26 (8.7)	6 (6.0)	0.395
	On respiratory support at baseline	57 (14.2)	31 (10.3)	26 (26)	0.000
	Time from onset of symptoms to hospital admission	5.75 ± 4.65	5.55 ± 4.27	6.36 ± 5.61	0.188
	Time from onset of symptoms to start of therapy	7.57 ± 4.89	7.25 ± 4.45	8.53 ± 5.93	0.052
	Early ≤ 7days	226 (56.6)	180 (60)	46 (46.5)	0.018
	Delayed >7 days	173 (43.4)	120 (40)	53 (53.5)	
	Duration of therapy (days)	13.03 (3.01)	13.01 (2.82)	13.08 (3.55)	0.848
**Vital signs**					
	Systolic BP	146.5 [26]	144 [24]	155 [34]	0.000
	Diastolic BP	92 [13]	91.0 [12]	94.5 [16]	0.192
	Pulse Rate	100 [23]	102.0 [23]	101.0 [30]	0.758
	Respiratory Rate	18 [2]	18.0 [2]	19.0 [4]	0.685
	Temperature	37.6 [1.4]	37.7 [1.3]	37.6 [1.4]	0.823
	Oxygen Saturation	97.0 [4]	97.0 [3]	97.0 [5]	0.036
**Laboratory findings**					
WBC (x10^9^/uL)		6.5 [2.9]	6.0 [3.1]	6.2 [3.3]	0.804
Lymphocytes (x10^9^/L)		1.4 [0.7]	1.2 [0.6]	1.1 [0.8]	0.115
Neutrophils (x10^9^/L)		4.2 [2.5]	4.2 [2.8]	4.4 [2.6]	0.889
CRP (mg/dL)		51.1 [52.2]	57.3 [88.4]	63.9 [93.0]	0.573
Procalcitonin (ng/ml)		0.2 [0.3]	0.3 [0.9]	0.4 [0.7]	0.005
D-Dimer (mg/L)		1.3 [3.3]	1.1 [1.0]	0.9 [1.2]	0.305
Ferritin (ug/L)		704.4 [720.7]	658.5 [777.3]	1011.0 [770.0]	0.006
Serum Creatinine (umol/L)		79.1 [20.0]	86.0 [22.0]	86.5 [29.0]	0.055
ALT (U/L)		49.5 [53.7]	35.0 [27.0]	30.0 [19.7]	0.147
AST (U/L)		36.0 [26]	38.0 [27]	38.5 [28]	0.933
**Radiological finding**					
	Bilateral Abnormalities	261 (65.3)	190 (63.3)	71 (71)	0.163
	Infiltration	142 (35.5)	118 (39.3)	24 (24)	0.006
	Ground glass Opacity	62 (15.5)	54 (18)	8 (8)	0.017
	Patchy Opacity	226 (56.5)	162 (54)	64 (64)	0.081
	Consolidation	89 (22.3)	61 (20.3)	28 (28)	0.110
	Location of Abnormality				0.043
	Upper	15 (3.8)	14 (4.7)	1 (1)	
	Middle	43 (10.8)	35 (11.7)	8 (8)	
	Lower	114 (36)	110 (36.7)	34 (34)	
	Upper-Middle	4 (1)	4 (1.3)	0 (0)	
	Lower-Middle	112 (28)	78 (26)	34 (34)	
	All Over	21 (5.3)	11 (3.7)	10 (10)	
	Had HRCT scan	33 (8.3)	25 (8.3)	8 (8)	0.916
	Had baseline ECG	382 (95.5)	291 (97.0)	91 (91.0)	0.012
	QTc Interval (ms)	425.8 ± 31.2	425.7 ± 30.5	426.3 ± 33.6	0.870
**Comorbidities**					
	No comorbidities	215 (53.8)	170 (56.7)	45 (45)	0.043
	DM	115 (28.7)	79 (26.3)	36 (36)	0.064
	HTN	106 (26.5)	69 (23)	37 (37)	0.006
	Dyslipidemia	43 (10.8)	24 (8.0)	19 (19)	0.002
	CKD (moderate to severe)	16 (4)	6 (2.0)	10 (10.0)	0.000
	MI	15 (3.8)	10 (3.3)	5 (5.0)	0.447
	COPD/Asthma	20 (5.0)	16 (5.3)	4 (4.0)	0.596
	Chronic liver disease (moderate to severe)	2 (0.5)	0 (0)	2 (2.0)	0.062
	Solid tumor	4 (1)	1 (0.3)	3 (3.0)	0.050
	CCI	0.59 ± 1.07	0.49 ± 0.85	0.91 ± 1.53	0.010
**Co-Medications**					
	Oseltamivir	400 (100)	300 (100)	100 (100)	NA
	Chloroquine/hydroxychloroquine	399 (99.8)	299 (99.7)	100 (100)	0.563
	Azithromycin	391 (97.8)	299 (99.7)	92 (92)	0.000
	B-lactam antibiotics	395 (98.8)	298 (99.3)	97 (97)	0.069
	Ribavirin	70 (17.5)	22 (7.3)	48 (48)	0.000
	Anticoagulants	359 (89.8)	266 (88.7)	93 (93)	0.216

Data presented as number (percentage), mean ± standard deviation, or median [interquartile range]

Abbreviations: WBC: White blood cells, CRP: C-Reactive Protein, ALT: Alanine Aminotransferase, AST: Aspartate Aminotransferase, HRCT: High-Resolution Computed Tomography, ECG: Electrocardiogram, DM: Diabetes mellitus, HTN: Hypertension, CKD: Chronic kidney disease, MI: Myocardial infarction, COPD: Chronic obstructive pulmonary disease, CCI: Charlson Comorbidity Index

Note: The total percentage is based on valid percent after considering for missing data; Independent t-test and Chi-square test were used at alpha level = 0.05

### Primary outcome

**Fig 1:**
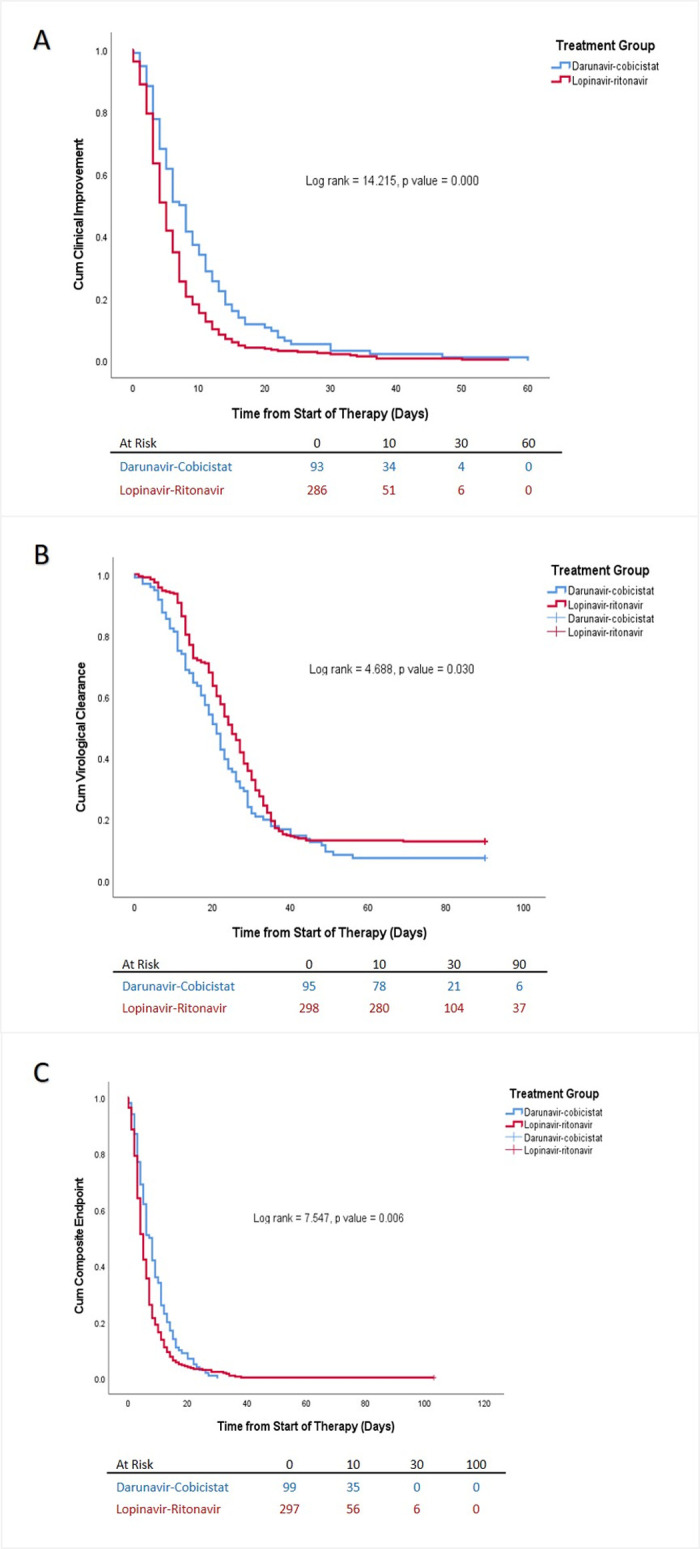
Kaplan-Meier curve for the time to primary outcomes. (A) Time to clinical improvement. (B) Time to viral clearance. (C) Time to first composite of primary outcome.

Patients in the lopinavir-ritonavir group had a significantly faster median time to clinical improvement and/or virological clearance than darunavir-cobicistat group (4 days [IQR 3–7] vs. 6.5 days [IQR 4–12]; HR 1.345 [95%CI: 1.070–1.691], P = 0.011). Patients in the lopinavir-ritonavir group had a significantly faster median time to clinical improvement than the darunavir-cobicistat group (5 days [IQR 3–8] vs. 8 days [IQR 4–13]; HR 1.520 (95%CI: 1.2–1.925), P = 0.000), while they have significantly slower time to virological clearance when compared with patients who received darunavir-cobicistat (25 days [IQR 15–33] vs. 21 days [IQR 12.8–30.0]; HR 0.772 (95%CI: 0.607–0.982), P = 0.035). Results of primary outcomes are presented in Fig 1 and Table 2.

**Table 2:** Results of the primary study outcomes.

Outcome	Total	Lopinavir-Ritonavir	Darunavir-Cobicistat	P-value	Log rank	P-value	HR	95% CI	P-value
Time to clinical improvement (days)	5 [3–9]	5 [3–8]	8 [4–13]	0.000	14.215	0.000	1.520	1.200–1.925	0.000
Time to virological clearance (days)	24 [14–33]	25 [15.0–33.0]	21.0 [12.8–30.0]	0.009	4.688	0.030	0.772	0.607–0.982	0.035
Time to composite primary outcome (days)	5 [3–9]	4 [3–7]	6.5 [4–12]	0.000	7.547	0.006	1.345	1.070–1.691	0.011

Data presented as median [Interquartile range]

Note: HR = Hazard ratio

### Adjustment for covariates

Due to the retrospective nature of the study, multiple confounders might contribute to the observed outcomes. Therefore, the Cox proportional-hazards model was used and adjusted for the statistically significant and clinically relevant baseline variables. Variables were limited to 10 factors to avoid over fitting the model. These factors include the region of origin, age, bilateral radiological abnormalities, infiltration, shortness of breath, time to start of therapy (early vs delayed), CCI of <1, hypertension, oxygen saturation >94% at baseline, and receiving ribavirin therapy.

The results of the primary outcomes after adjustments for covariates were summarized in the supporting information section S1 Table. The Cox’s proportional hazards models for the three outcome measures were significant (p < 0.01). The Kaplan-Meier analysis on the time to primary outcomes was used to compare survival curves using log rank test. The findings indicated that there were significant differences in the survival curves for different covariates.

### Secondary outcomes

Table 3 illustrates the results of the secondary outcomes. For the percentage of virological clearance, more patients in the darunavir-cobicistat group had significantly achieved virological clearance at day 21 and day 28 when compared to patients in the lopinavir-ritonavir group. However, the difference in virological clearance was not significant on day 14. Furthermore, third of the patients who received darunavir-cobicistat clinically deteriorated after two days of therapy, mainly due to the need for corticosteroids/immunomodulation therapy and the need for respiratory support.

**Table 3:** Results of the secondary outcomes.

Outcome		Total	Lopinavir-Ritonavir	Darunavir-Cobicistat	P value
	Virological clearance at day 14	113 (28.2)	79 (26.3)	34 (34)	0.140
	Virological clearance at day 21	176 (44)	123 (41)	53 (53)	0.036
	Virological clearance at day 28	256 (64)	183 (61)	73 (73)	0.030
	Clinical deterioration (composite)	100 (25)	66 (22)	34 (34)	0.016
	Need for respiratory support	53 (13.3)	39 (13)	14 (14)	0.798
	Vasopressor use	17 (4.3)	10 (3.3)	7 (7)	0.102
	Corticosteroids/ immunomodulation use	62 (15.5)	39 (13)	23 (23)	0.017
	Prone positioning	29 (7.2)	19 (6.3)	10 (10)	0.221
	Development of acute respiratory distress syndrome	76 (19.0)	46 (15.3)	30 (30.0)	0.001
	Length of hospital stay	13.71 (17.8)	12.04 (19.8)	15.26 (11.5)	0.001
	All-cause mortality at 30-days	5 (1.3)	0 (0)	5 (5.0)	0.001

Data presented as number (percentage)

Note: The total percentage is based on valid percent after considering for missing data; Mann-Whitney test and Chi-square/Fisher’s Exact test were used at alpha level = 0.05

Fewer patients in the lopinavir-ritonavir group developed ARDS when compared to patients who received darunavir-cobicistat (p = 0.001). Furthermore, the length of hospital stay was significantly shorter for patients in lopinavir-ritonavir treatment (p = 0.015). All-cause mortality at day 30 was significantly lower in the lopinavir-ritonavir group when compared to the darunavir-cobicistat group (p = 0.001).

### Safety outcomes

The difference between the two treatment arms in term of incidence of adverse events were not significant except for QTc interval prolongation Table 4. More patients in the darunavir-cobicistat group had prolonged QTc interval > 500 ms (13% vs 2.7%, p = 0.000). Twenty-four ADRs occurred in the lopinavir-ritonavir group, which were mainly due to elevated liver transaminase levels. The majority of reported ADRs were of grade 1 and grade 3 (11 (2.8%) and 10 (2.5%), respectively). The rate of premature therapy discontinuation was not different among both groups.

**Table 4:** Safety outcomes of the study population.

Outcome	Total	Lopinavir-Ritonavir	Darunavir-Cobicistat	P value
**Incidence of adverse events**	28 (7.2)	23 (8.0)	5 (5.0)	0.316
**Type of ADR:**				0.219
**Elevated liver transaminase levels**	21 (5.3)	19 (6.3)	2 (2.0)	
**PR prolongation**	1 (0.3)	1 (0.3)	0 (0)	
**Renal impairment**	5 (1.3)	2 (0.7)	3 (3.0)	
**Neutropenia**	1 (0.3)	1 (0.3)	0 (0)	
**QTc interval prolongation**				
**QTc prolongation > 500**	21 (5.3)	8 (2.7)	13 (13.0)	0.000
**QTc prolongation > 550**	7 (1.8)	3 (1.0)	4 (4.0)	0.048
**Grade of ADR**				0.749
**Grade 1**	11 (2.8)	9 (3.0)	2 (2.0)	
**Grade 2**	7 (1.8)	5 (1.7)	2 (2.0)	
**Grade 3**	10 (2.5)	9 (3.0)	1 (1.0)	
**Grade 4**	1 (0.3)	1 (0.3)	0 (0)	
**Time to ADRs development**	9.0 [3.5]	9.0 [3]	10.0 [3.0]	0.114
**Rate of premature discontinuation of study treatment**	70 (17.5)	51 (17.0)	19 (19.0)	0.649
**Reason for premature discontinuation**				0.658
**ADR**	29 (7.2)	24 (8.0)	5 (5.0)	
**Drug interaction**	3 (0.8)	2 (0.7)	1 (1.0)	
**Others**	73 (18.3)	52 (17.3)	21(21.0)	

## Discussion

This multicenter observational study was the first study to compare the efficacy and safety outcomes of two protease inhibitors used to treat COVID-19 infection. In this study, we found that in hospitalized patients with COVID-19 pneumonia, early treatment with lopinavir-ritonavir (within seven days of symptoms onset) in addition to standard of care is associated with a significantly shorter time to clinical improvement and/or virological clearance when compared to treatment with darunavir-cobicistat therapy. The observed effect was mainly attributed to significantly shorter time to clinical improvement (P = 0.000). On the other hand, treatment with lopinavir-ritonavir was associated with a significantly longer time to virological clearance. These results were consistent after adjusting for possible covariates.

Our patient population was heterogenous at baseline in terms of severity of the disease and duration of antiviral therapy compared to previously published studies that evaluated the effect of protease inhibitors in COVID-19 separately [16, 17, 20, 33]. After the positive effect of lopinavir-ritonavir use in a COVID-19 patient with mild symptoms in Korea [16], authors recommended its use from the early stage of infection. However, subsequent controlled studies used lopinavir-ritonavir in patients with more severe disease and after seven days of symptoms onset [17, 33]. B.Cao et al. studied the effect of lopinavir-ritonavir is severe COVID-19 infection in which all study population were in respiratory distress at baseline with a median time from symptoms onset to start of therapy of 13 days [17]. Additionally, 74% of patients in the RECOVERY trial required respiratory support at baseline and treatment was started within eight days of symptoms onset, which could have contributed to the negative effect of the treatment in both studies [33]. It is important to note the importance of early initiation of antiviral therapy during the viral replication phase of COVID-19 pathogenesis over the host inflammatory response phase, which can be translated into the lack of clinically significant anti-SARS-CoV-2 activity if used in late or severe stages of the disease [34]. This hypothesis was also emphasized in the National Institutes of Health COVID-19 Treatment Guideline where the role of antiviral medications in treating mild, moderate, severe, and critical illness was stressed in order to optimize the treatment for people with COVID-19 [A]. In our study, only 14.2% of the population had the severe disease at baseline and the median time from symptoms onset to start therapy was approximately 7 days, which could contribute to the significant effect observed.

Treatment with darunavir-cobicistat was associated with faster virological clearance and higher rate of negative conversion of SARS CoV-2 at day 21 and day 28 compared to lopinavir-ritonavir. These findings are in line with previous evidence showing that the median duration of COVID-19 viral shedding in patients with mild-moderate disease is 20 days [35].

Protease inhibitors are mainly used for the treatment of HIV infection by binding to the HIV-1 protease activity site. This led to the inhibition of the viral Gag-Pol polyprotein precursors cleavage into individual functional proteins, resulting in a noninfectious, immature viral particles [36]. In fact, the target protease enzymes involved by HIV and SARS-CoV-2 are different, as the HIV protease is an aspartic protease, whereas SARS-CoV-2 is a cysteine protease [37]. Both darunavir/cobicistat and lopinavir/ritonavir were proposed as a candidate therapies for COVID-19 as they inhibit the enzymes that activate envelope glycoproteins as part of the viral entry process. Furthermore, Both drugs have been shown to bind well to the SARS-CoV 3C-like protease (3CLpro), which is involved in the viral replication process [38]. Nevertheless, they are likely to behave differently in the treatment of COVID-19 patients and also to display different side effects. In some articles lopinavir was found to have a higher theoretical affinity for SARS-CoV-2 3CLpro than that of darunavir [39, 40], while others showed that darunavir has larger binding free energies to SARS-CoV-2 3CLpro [41–43]. Therefore, the exact mechanism by which these drugs may contribute to virological clearance of SARS-CoV-2 remains to be elucidated.

Additionally, our study showed that patients in the darunavir-cobicistat group had more clinical deteriorations, more incidence of ARDS, and all-cause mortality at day 30. However, it is unclear whether the observed difference is due to the antiviral therapy or the concomitant medications (ex. ribavirin) or the baseline clinical status of the patients. In a recent retrospective report conducted in Qatar, the use of darunavir-cobicistat plus ribavirin was associated with a more complicated course in term of the need for ICU admission, intubation, and progression to ARDS [44]. Furthermore, it is important to note that patients who received darunavir-cobicistat had older age, more comorbidities, and more severe disease at baseline. Therefore, these findings are mainly hypothesis-generating and need to be confirmed in well-conducted randomized trials.

The overall mortality rate in our study was very low (1.3%), which is substantially lower than the mortality reported in previous studies (20–23%) [17, 33]. This indicates the milder disease the patients had and reflects the relatively low mortality rate in the country.

The safety profile in this study was somehow consistent with the previous studies. Our study is the first one that assessed the effect of protease inhibitors on QTc interval prolongation [17, 33]

Both drugs were well-tolerated, and the majority of the ADRs that led to premature treatment discontinuation were of grade 1 and were due to the elevation of liver transaminases.

Our study, which is the first to compare the clinical, laboratory, virological, and radiological outcomes of two protease inhibitors in COVID-19, has several limitations. First, the retrospective observational nature of the study. Multivariate analyses were used to evaluate the association of possible covariates on study outcomes. Additionally, only adverse events that led to treatment discontinuation were reported, and details about side effects (ex. gastrointestinal side effects) occurring during the treatment course were not collected. These side effects are well-known and have been studied and reported in previous studies [17].

## Conclusion

In hospitalized patients with COVID-19 pneumonia, early treatment with lopinavir-ritonavir was associated with significantly faster time to clinical improvement and/or virological clearance than darunavir-cobicistat. Treating patients with lopinavir-ritonavir resulted in a faster time to clinical improvement, while treating patients with darunavir-cobicistat resulted in a faster clearance of the virus. The safety profile of both protease inhibitors was comparable, with more incidence of QTc interval prolongation, ARDS development, clinical deterioration, and mortality in darunavir-cobicistat group. Future prospective trials are warranted to confirm these findings.

## Supporting information

S1 Table Results of primary outcome after adjustments for covariates. (DOCX)

S1 Dataset Minimal data set. (XLSX)
