# Supplementary material for: Darunavir-cobicistat versus lopinavir-ritonavir in the treatment of COVID-19 infection (DOLCI): A multicenter observational study
Source: PLoS One. 2022 May 4;17(5):e0267884. doi: 10.1371/journal.pone.0267884 (PMC9067693; doi:10.1371/journal.pone.0267884)
Supplement: S1 Table — (DOCX) [file pone.0267884.s001.docx]

**Supporting Information**

**Darunavir-Cobicistat versus Lopinavir-Ritonavir in the Treatment of COVID-19 Infection (DOLCI): A Multicenter Observational Study**

This appendix has been provided by the authors to give readers additional information about their work.

**Table of Content**

Adjustment for covariates ……………………………………………………………………….. 3

Appendix Table …………………………………………………………………………………..5

***S1 Text. Adjustment for Covariates***

Cox's proportional hazards model for analyzing time to clinical improvement was found significant. The p values indicate that there is a strong evidence that patients with bilateral radiological abnormalities, having shortness of breath, started on antiviral therapy after 7 days of symptoms onset, or received ribavirin therapy were associated with longer time to clinical improvement.

For the time to virological clearance, the Cox's proportional hazards model was also found significant. The p value indicates that there is a strong evidence that the region of origin is associated with longer time to virological clearance, while receiving antiviral therapy after 7 days of symptoms onset was bordering on statistical significance.

The p values for Cox regression analysis for the composite primary endpoint of clinical and/or virological improvement indicate that there is a strong evidence that having bilateral radiological abnormalities and having shortness of breath are associated with longer time to composite of primary outcomes.

The Kaplan-Meier analysis on time to clinical improvement was used to compare survival curves using log rank test. The findings indicated that there was a significant difference of the survival curves between the region of origin, age, bilateral radiological abnormalities, shortness of breath, delayed onset of antiviral therapy treatment, CCI of <1, hypertension, oxygen saturation of > 94% at baseline, and receiving ribavirin therapy.

The Kaplan-Meier analysis on time to virological clearance was used to compare survival curves using log rank test. The findings indicated that there was significant difference of the survival curves between the region of origin, age, and delayed onset of antiviral therapy.

The time to composite of primary outcomes was censored. The Kaplan-Meier analysis indicated that there was a significant difference of the survival curves between the bilateral radiological abnormalities, shortness of breath, CCI of <1, oxygen saturation of > 94% at baseline, and receiving ribavirin therapy. The p value of the difference between having or not having hypertension was bordering on statistical significance.

Further, multivariate regression analysis was carried out to remove the effect of confounders by bringing statistically and clinically significant covariates into the model. The statistical output demonstrated that the regression model is significant (adjusted R squared = 12.4%, F=4.956, p = less than 0.001). Durbin-Watson was also significant (DW=2, p = less than 0.001) which indicated that the modelling is good and has no autocorrelation. Two covariates i.e. shortness of breath (p= 0.003, beta=3.184, 95% CI for beta: 1.086 – 5.283) and the need for respiratory support (p=0.036, beta=3.038, 95% CI for beta: 0.207 – 5.870) were significant predictors for the patient’s clinical improvement. The model was further established by considering the treatment groups (Kaletra as reference) as selection variable to limit the analysis to a subset of cases having a particular value(s) for this variable. Again the two covariates were significant predictors i.e. shortness of breath (p= 0.034, beta=2.410, 95% CI for beta: 0.183 – 4.637) and the need for respiratory support (p=0.005, beta=4.398, 95% CI for beta: 1.368 – 7.429). There was no autocorrelation and modelling is proven fit for the covariates (DW=1.938, p =0.006). ANOVA analysis indicated that the model is significant (adjusted R squared = 7.3%, F=2.810, p = 0.006).

**S1 Table. Results of primary outcome after adjustments for covariates**

|  | Variables | Log rank (chi-square test) | P-value | Adjusted Hazard ratio | 95% CI | | P-value |
| --- | --- | --- | --- | --- | --- | --- | --- |
| Time to Clinical Improvement | |  |  |  |  |  | 0.000 |
|  | Region of Origin | 11.271 | 0.024 |  |  |  | 0.432 |
|  | America |  |  | 1.013 | 0.403 | 2.545 | 0.978 |
|  | East Africa |  |  | 0.625 | 0.357 | 1.094 | 0.100 |
|  | Europe |  |  | 1.046 | 0.488 | 2.242 | 0.907 |
|  | Middle East |  |  | 0.838 | 0.619 | 1.135 | 0.254 |
|  | South Asia (Ref) |  |  | Ref |  |  |  |
|  | Age | 10.407 | 0.001 | 0.795 | 0.576 | 1.098 | 0.164 |
|  | Bilateral abnormalities | 22.608 | 0.000 | 0.735 | 0.584 | 0.926 | 0.009 |
|  | Infiltration | 1.665 | 0.197 | 1.120 | 0.898 | 1.396 | 0.314 |
|  | Dyspnea | 40.887 | 0.000 | 0.624 | 0.497 | 0.783 | 0.000 |
|  | Early vs Delayed | 8.795 | 0.003 | 1.476 | 1.193 | 1.826 | 0.000 |
|  | No comorbidities | 7.107 | 0.008 | 0.964 | 0.728 | 1.277 | 0.800 |
|  | HTN | 6.888 | 0.009 | 0.984 | 0.715 | 1.354 | 0.919 |
|  | SpO2 > 94% at baseline | 19.411 | 0.000 | 1.198 | 0.853 | 1.683 | 0.296 |
|  | Ribavirin Therapy | 36.870 | 0.000 | 0.595 | 0.425 | 0.835 | 0.003 |
| Time to Virological Clearance | |  |  |  |  |  | 0.003 |
|  | Region of Origin | 21.047 | 0.000 |  |  |  | 0.016 |
|  | America |  |  | 1.167 | 0.465 | 2.925 | 0.742 |
|  | East Africa |  |  | 0.876 | .473 | 1.622 | 0.673 |
|  | Europe |  |  | 2.656 | 2.656 | 1.278 | 0.009 |
|  | Middle East |  |  | 1.455 | 1.455 | 1.085 | 0.012 |
|  | South Asia (Ref) |  |  | Ref |  |  |  |
|  | Age | 5.759 | 0.016 | 1.174 | .857 | 1.607 | 0.318 |
|  | Bilateral abnormalities | 0.581 | 0.446 | 0.852 | .667 | 1.090 | 0.202 |
|  | Infiltration | 3.087 | 0.079 | 0.910 | .722 | 1.147 | 0.425 |
|  | Dyspnea | 0.451 | 0.502 | 0.913 | .725 | 1.150 | 0.440 |
|  | Early vs Delayed | 4.193 | 0.041 | 1.249 | 1.003 | 1.554 | 0.047 |
|  | No comorbidities | 0.162 | 0.687 | 1.042 | 0.778 | 1.394 | 0.784 |
|  | HTN | 0.101 | 0.750 | 1.053 | 0.764 | 1.453 | 0.751 |
|  | SpO2 > 94% at baseline | 1.178 | 0.278 | 1.303 | 0.929 | 1.828 | 0.125 |
|  | Ribavirin Therapy | 1.427 | 0.232 | 1.200 | 0.854 | 1.688 | 0.294 |
| Time to Composite Outcome | |  |  |  |  |  | 0.000 |
|  | Region of Origin | 5.508 | 0.239 |  |  |  | 0.758 |
|  | America |  |  | 1.082 | 0.433 | 2.703 | 0.866 |
|  | East Africa |  |  | 0.788 | 0.450 | 1.379 | 0.404 |
|  | Europe |  |  | 0.859 | 0.421 | 1.754 | 0.677 |
|  | Middle East |  |  | 0.853 | 0.641 | 1.137 | 0.279 |
|  | South Asia (Ref) |  |  | Ref |  |  |  |
|  | Age | 3.188 | 0.074 | 1.003 | 0.746 | 1.349 | 0.982 |
|  | Bilateral abnormalities | 20.416 | 0.000 | 0.697 | 0.557 | 0.872 | 0.002 |
|  | Infiltration | 0.597 | 0.440 | 1.117 | 0.895 | 1.394 | 0.327 |
|  | Dyspnea | 25.985 | 0.000 | 0.699 | 0.560 | 0.871 | 0.001 |
|  | Early vs Delayed | 3.011 | 0.083 | 1.226 | 0.995 | 1.511 | 0.056 |
|  | No comorbidities | 4.71 | 0.030 | 1.062 | 0.807 | 1.396 | 0.669 |
|  | HTN | 3.915 | 0.048 | 1.025 | 0.755 | 1.391 | 0.875 |
|  | SpO2 > 94% at baseline | 13.848 | 0.000 | 1.302 | 0.942 | 1.800 | 0.110 |
|  | Ribavirin Therapy | 15.761 | 0.000 | 0.872 | 0.628 | 1.209 | 0.410 |
| Abbreviations: HTN: Hypertension  Note: Cox regression analysis and Log rank test were done at alpha level = 0.05 | | | | | | | |
